# Supplementary material for: Structurally distinct telomere-binding proteins in Ustilago maydis execute non-overlapping functions in telomere replication, recombination, and protection
Source: Commun Biol. 2020 Dec 16;3:777. doi: 10.1038/s42003-020-01505-z (PMC7744550; doi:10.1038/s42003-020-01505-z)
Supplement: Supplementary file 2 — Supplementary Information [file 42003_2020_1505_MOESM2_ESM.pdf]

Structurally distinct telomere-binding proteins in *Ustilago maydis* execute non-overlapping functions in telomere replication, recombination and protection

Eun Young Yu[1], Syed Zahid[1], Swapna Ganduri[1], Jeanette Sutherland[1], Min Hsu[1], William K. Holloman[1], Neal F. Lue [1, 2] *

Supplementary Figures 1-12

Supplementary Tables 1-2

Supplementary Figure 13: Original Gel and Blot images

Supplementary Fig. 1

a

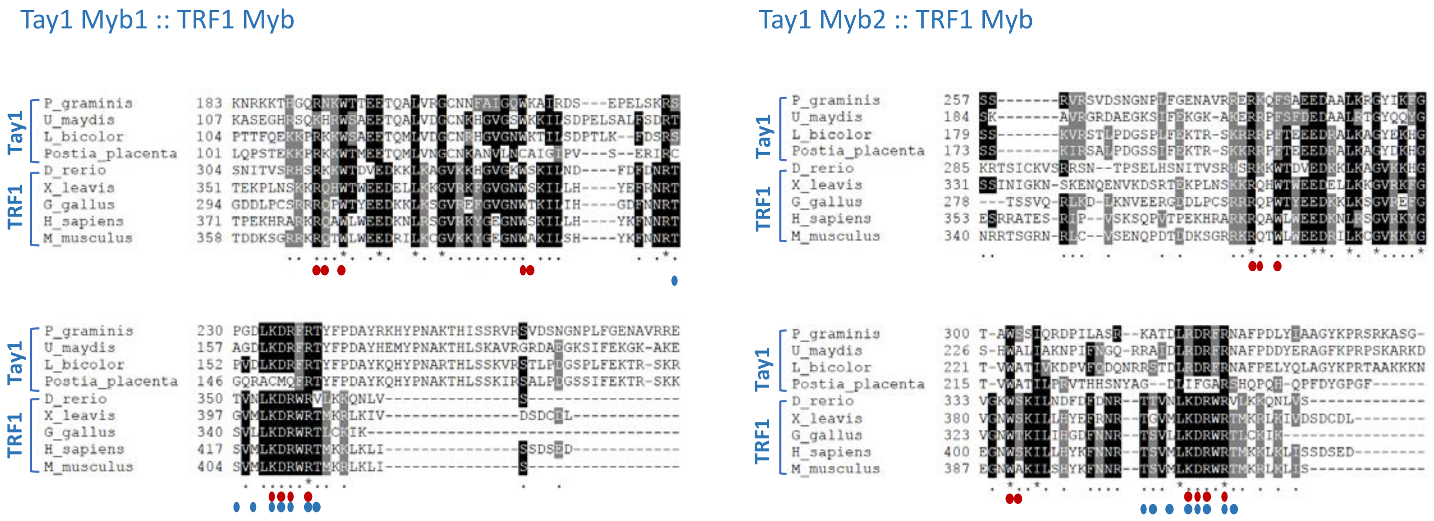

b

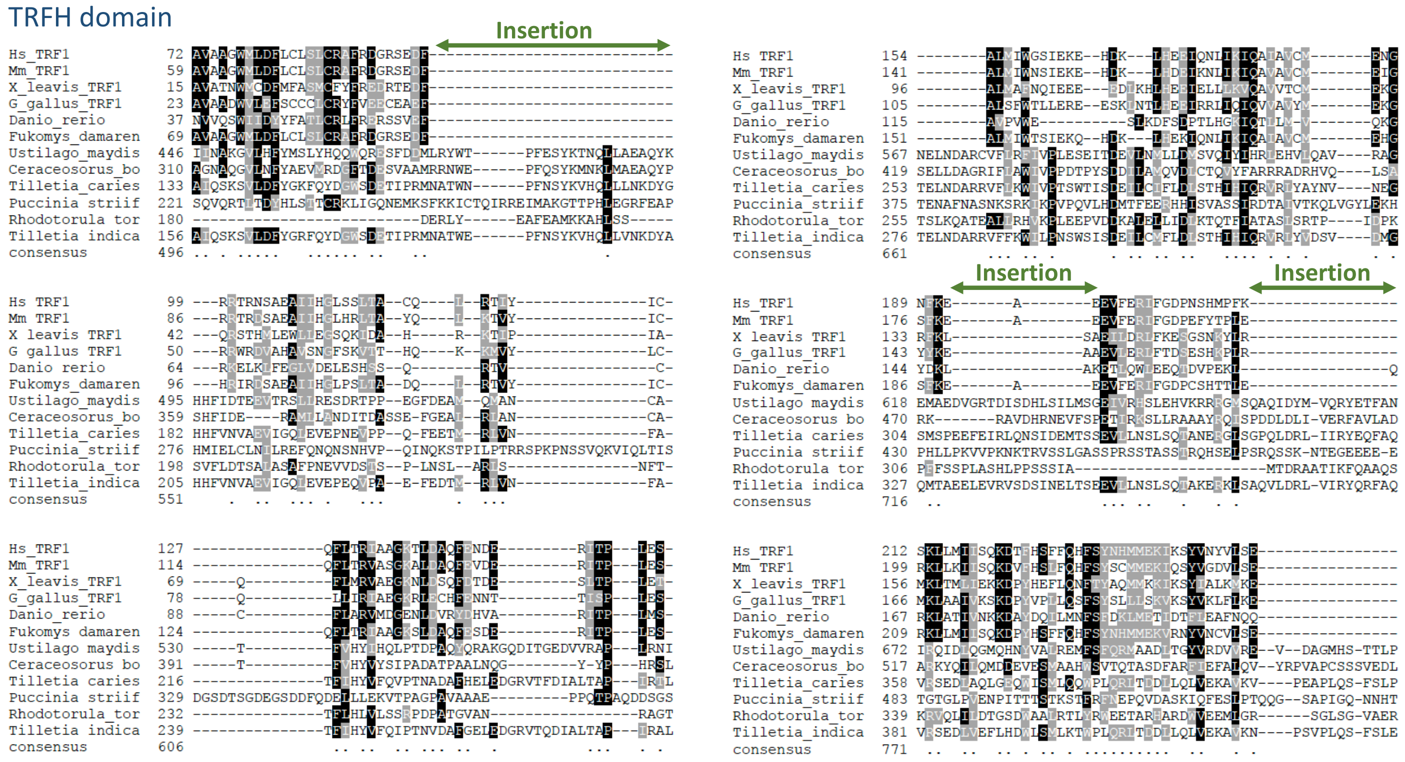

c

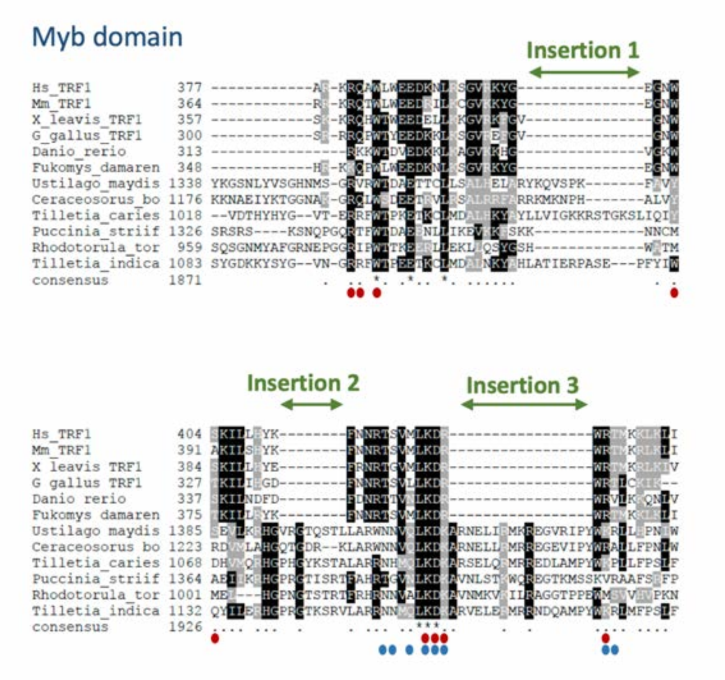

Supplementary Fig. 1. Alignments of Myb1 and Myb2 of Tay1 proteins to the Myb domains of metazoan TRF1s 1; Multiple sequence alignments of the TRFH and Myb domain in metazoan TRF1s and the UmTrf2 family of proteins

(a) The first and second Myb domains of Tay1 family members were aligned to metazoan TRF1 Myb domains. The homologs include: P_graminis, *Puccinia graminis*; U_maydis, *Ustilago maydis*; L_bicolor, *Laccaria bicolor*; Postia_placenta, *Postia placenta*; D_rerio, *Danio rerio*; X_leavis, *Xenopus leavis*; G_gallus, *Gallus gallus*; H_sapiens, *Homo sapiens*; M_musculus, *Mus musculus*. Residues predicted to make direct contacts to DNA and to make water-mediated contacts are highlighted in red and cyan, respectively (Court et al., 2005). **(b)** Full length proteins were aligned using T-coffee and the alignment for TRFH region is displayed. The homologs include: Hs_TRF1, from *H. sapiens*; Mm_TRF1, from *M. musculus*; X_leavis_TRF1, from *X. leavis*; G_gallus_TRF1; from *G. gallus*; Danio_rerio, from *D. rerio*; Fukomys_damaren; from *F. damarensis*; Ustilago_maydis; from *U. maydis* (UMAG_02458); Ceraceosorus_bo, from *C. bombacis* (CEH14748); Tilletia_caries, from *T. caries* (OAJ23777); Puccinia_striif, from *P. striiformis* (KNF05609); Rhodotorula_tor, from *toruloides* (XP_016271506); Tilletia_indica, from *T. indica* (OAJ06516). Three regions of insertions in the fungal proteins are highlighted. **(c)** Same as in **b** except that the Myb domain alignment is displayed. The three regions of insertions in the fungal proteins are highlighted. Residues predicted to make direct contacts to DNA and to make water-mediated contacts are highlighted in red and cyan, respectively (Court et al., 2005).

Supplementary Fig. 2

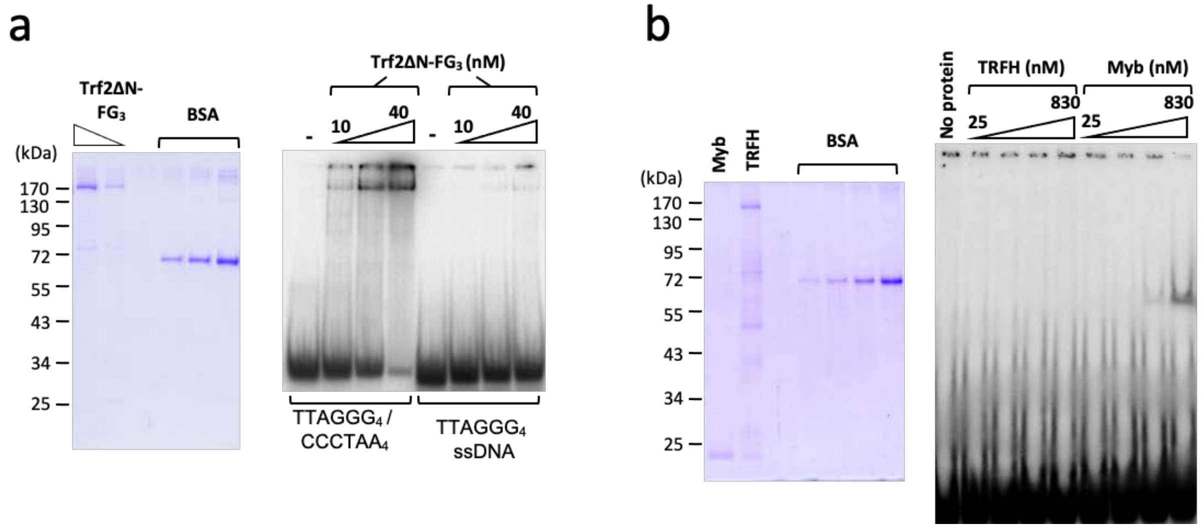

Supplementary Fig. 2. *Um*Trf2 recognizes double stranded telomere repeats
(a) (Left) Purified Trf2ΔN-FG_3 was subjected to SDS-PAGE and Coomassie staining along with BSA standards (0.12, 0.25 and 0.5 µg). (Right) EMSA analysis was performed using varying concentrations of Trf2ΔN-FG_3 (10, 20 and 40 nM) and either single-stranded or double-stranded telomere oligonucleotides. **(b)** (Left) Purified Trf2 Myb and TRFH domains were subjected to SDS-PAGE and Coomassie staining along with BSA standards (0.06, 0.12, 0.25, and 0.5 µg) . (Right) EMSA analysis was performed using varying concentrations of TRFH and Myb domains (25, 83, 250, and 830 nM) and double strand $TTAGGG_4$ probe.

Supplementary Fig. 3

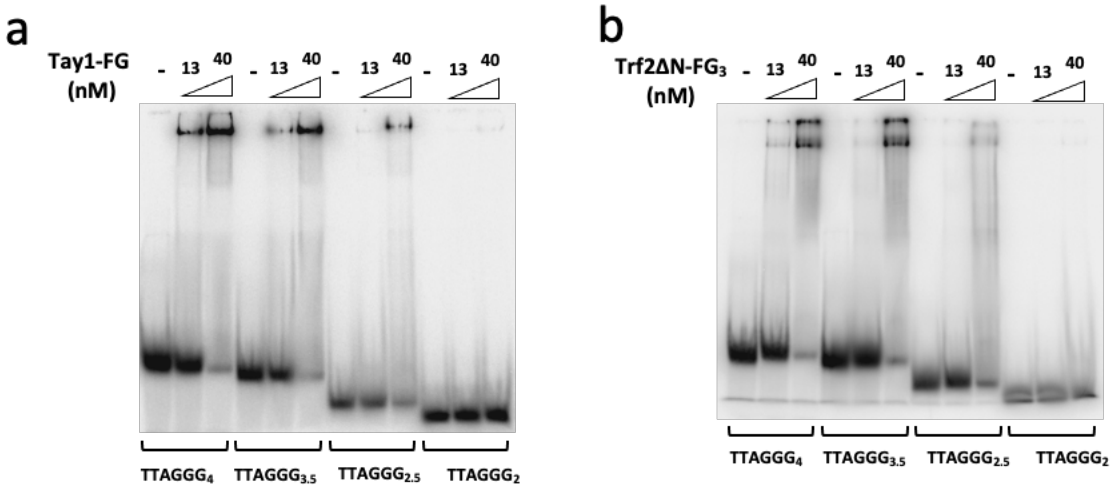

**$TTAGGG_4$**	TTA <u>GGGTTA</u> <u>GGGTTA</u> <u>GGGTTA</u> GGG
**$TTAGGG_{3.5}$**	TTA <u>GGGTTA</u> <u>GGGTTA</u> <u>GGGTTA</u> G
**$TTAGGG_{2.5}$**	TTA <u>GGGTTA</u> <u>GGGTTA</u> G
**$TTAGGG_2$**	GGTTA <u>GGGTTA</u> G

Supplementary Fig. 3. Analysis of the minimal DNA target site for *Um*Tay1 and *Um*Trf2
(a) EMSA was performed using 5 nM of the telomere probes and the indicated concentrations (in nM) of Tay1-FG.
(b) Same as in A except that Trf2ΔN-FG_3 was used instead of Tay1-FG. The sequences of the DNA probes used for these assays are displayed at the bottom.

Supplementary Fig. 4

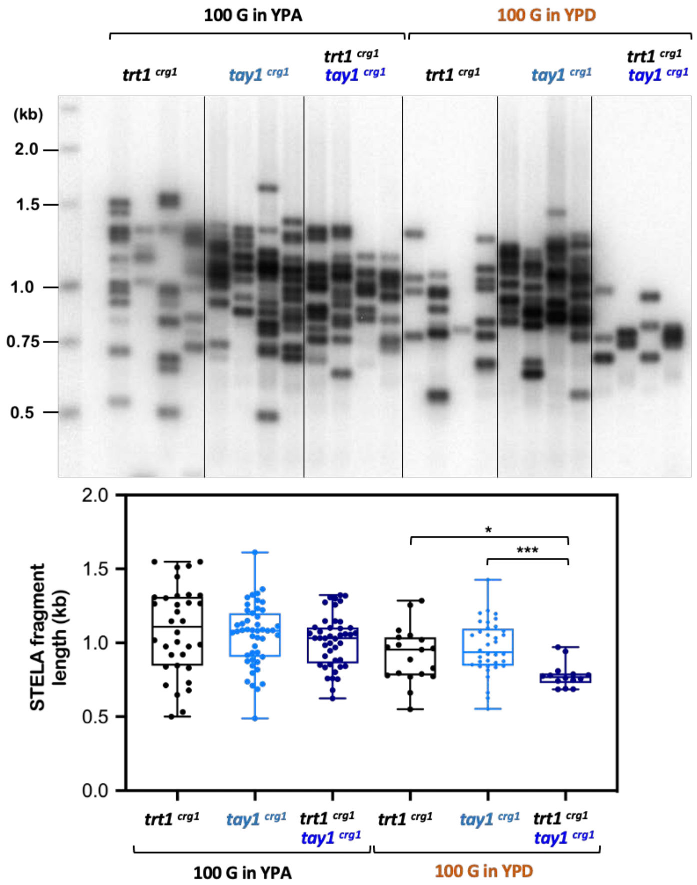

Supplementary Fig. 4. Transcriptional repression of tay1and trt1 results in accelerated telomere shortening

(Top) Conditional strains in which the expression of *tay1*, *trt1*, or both are controlled by the arabinose-dependent *crg1* promoter were constructed and propagated in permissive medium (YPA) or repressing medium (YPD) for 100 generations. The DNAs were isolated from the strains and subjected to STELA analysis.

(Bottom) The STELA PCR product lengths were calculated using TeSLA software and plotted to indicate range (top and bottom error bar), quartile (box), and average (middle bar).

Supplementary Fig. 5

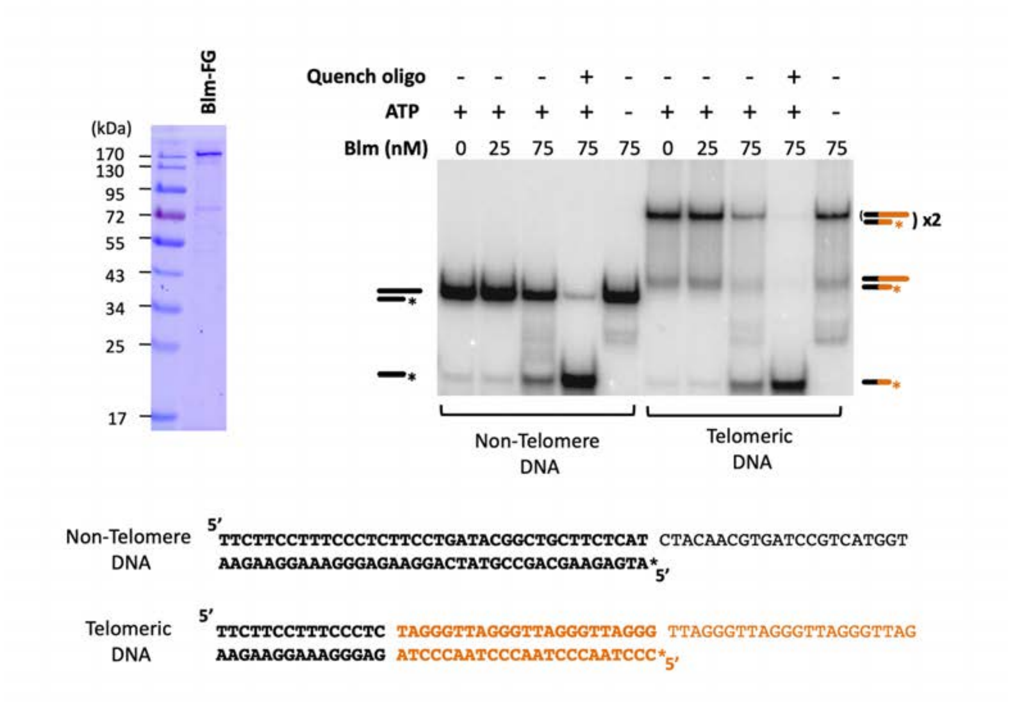

Supplementary Fig. 5. Unwinding activity of *U. maydis* Blm helicase on telomeric and non-telomeric DNA
(Top left) Purified Blm-FG was subjected to SDS-PAGE and Coomassie staining. (Top right) Helicase assays were performed using the indicated substrates and varying concentrations of Blm in the absence or presence of ATP. The reaction mixtures were analyzed by native gel electrophoresis and PhosphorImager scanning. Labeled species that correspond to dsDNA substrates and ssDNA products are designated schematically to the left and right of the gel. Asterisks indicate the positions of the P^{32} label. (Bottom) The sequences of the substrates are displayed with the non-telomeric and telomeric regions shown in black and brown, respectively.

Supplementary Fig. 6

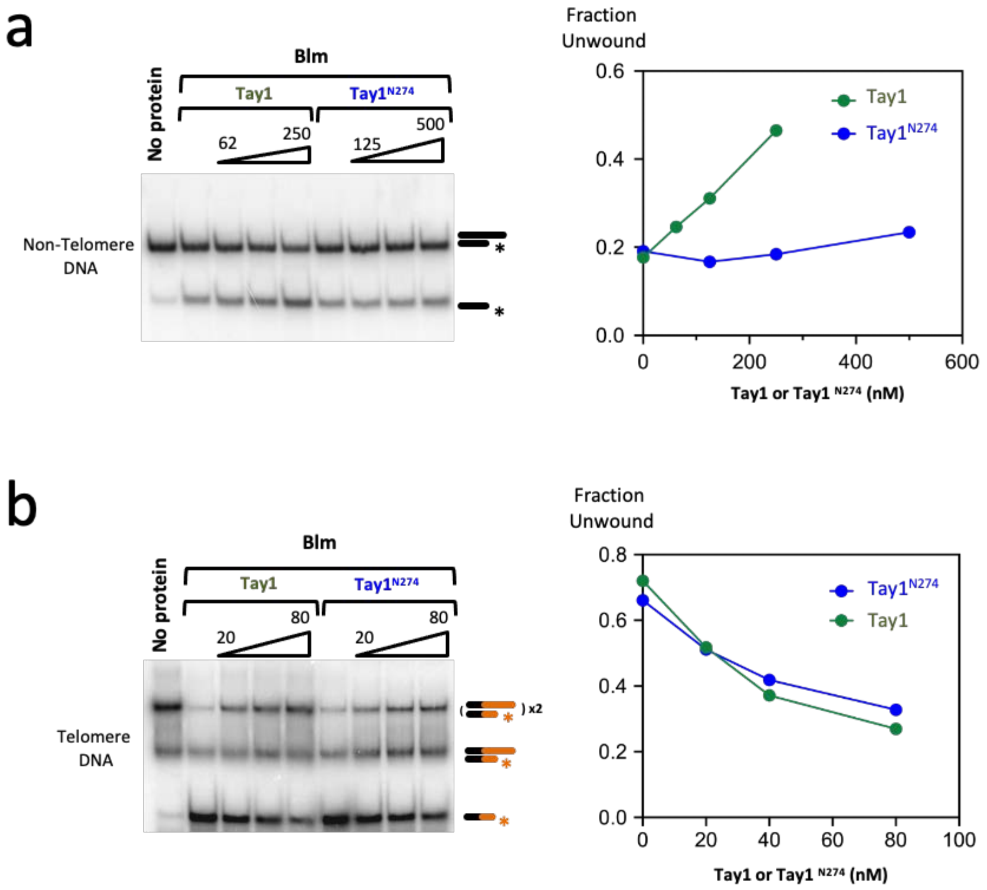

Supplementary Fig. 6. The effects of Tay1 and Tay1^{N274} on Blm helicase activity

(a) The helicase activity of Blm on non-telomeric DNA was examined in the presence of increasing concentrations of either Tay1 or Tay1^{N274}. The assay scan is shown on the left and the quantitation plotted on the right. **(b)** Same as in A except that assays were performed using the telomeric substrate.

Supplementary Fig. 7

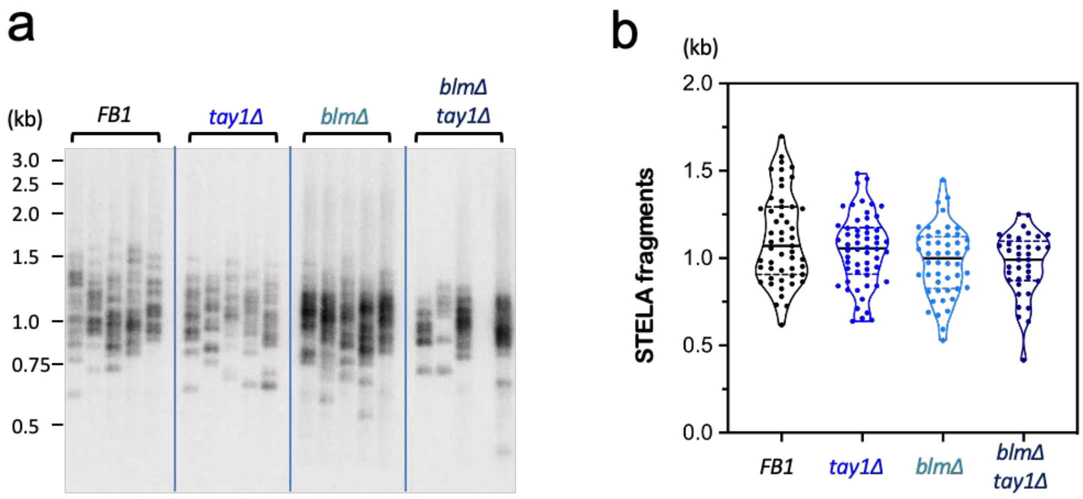

Supplementary Fig. 7. STELA analysis of *U. maydis* telomere replication mutants
(a) STELA assays for the indicated strains were performed in quintuplet. The amplified telomere fragments were analyzed by Southern using a probe that spans a region of the UT4/UT5 subtelomeric element. **(b)** The sizes of the STELA fragments in A were analyzed using the TeSLA program (Lai et al., 2017) and the results shown as violin plots.

Supplementary Fig. 8

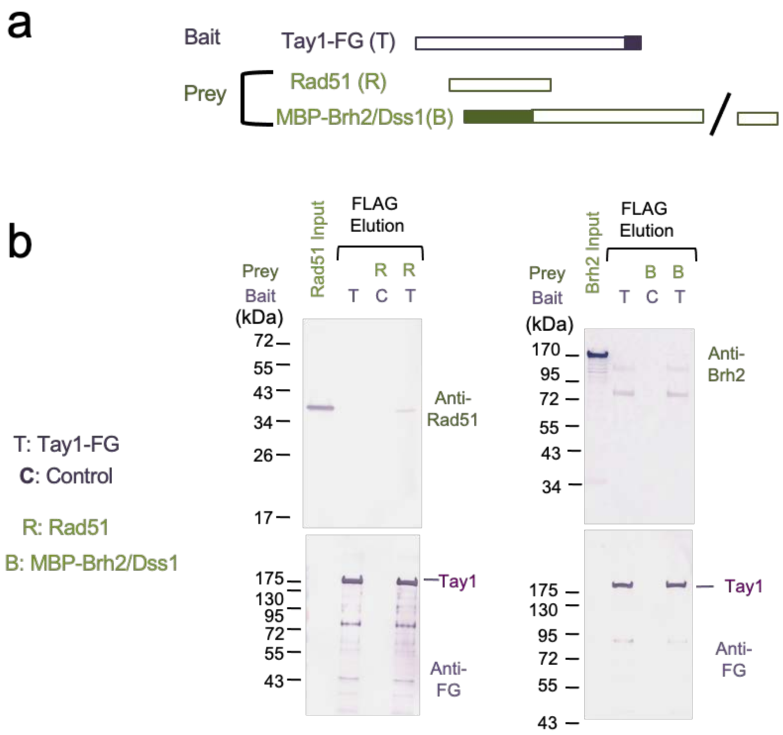

Supplementary Fig. 8. Analysis of potential interaction between Tay1 and either Rad51 or Brh2/Dss1
(a) The Bait and prey used in the pull down assays are schematically illustrated. Fill boxes indicate the locations of the affinity/epitope tags. **(b)** Tay1-FG was immobilized on FLAG beads and incubated with either Rad51 or the MBP-Brh2/Dss1 complex (~1 μM). Following incubation, the beads were washed and then eluted with FLAG peptide. The levels of the bait and prey proteins in the elution samples were analyzed by Western using appropriate antibodies. Neither Rad51 nor Brh2 could be detected in the pull down samples, suggesting that they do not interact strongly with Tay1.

Supplementary Fig. 9

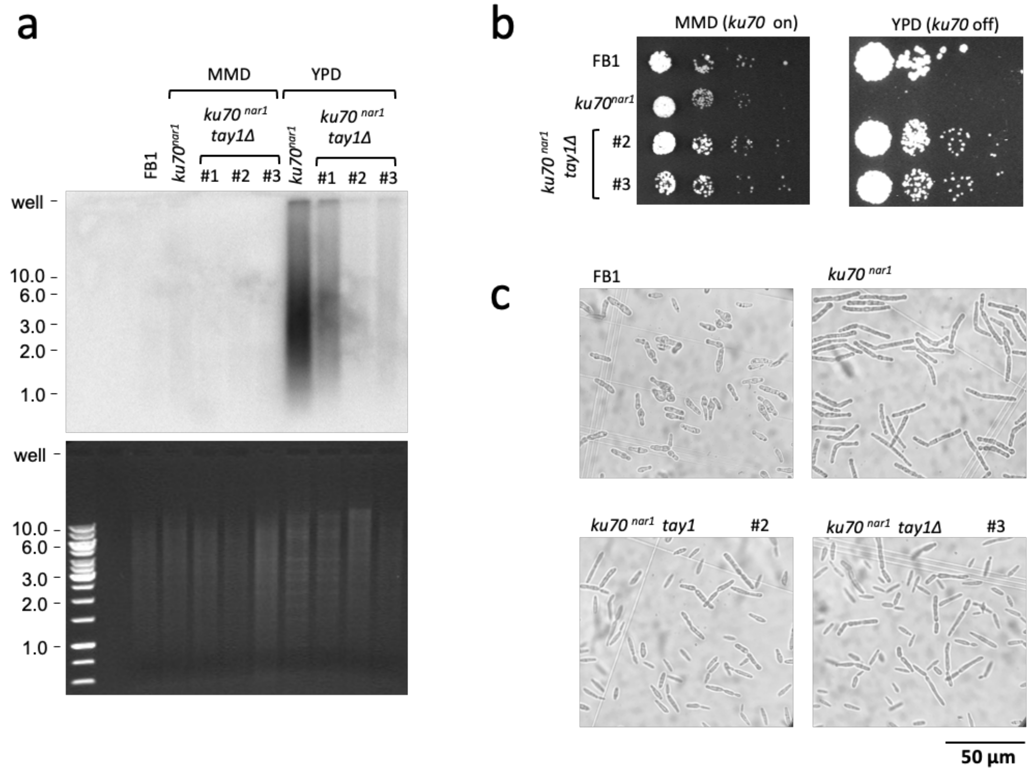

Supplementary Fig. 9. The effects of *tay1* deletion on C-strand ssDNA, as well as the growth and morphology of *ku70^{nar1}* mutant.

(a) The levels of C-strand ssDNA in the indicated strains grown in MMD and YPD were analyzed by in-gel hybridization (top). The ethidium bromide stained gel shows that similar amounts of DNAs were analyzed (bottom). **(b)** Two independently constructed *ku70^{nar1} tay1*Δ clones were analyzed for growth on MMD and YPD along with the parental FB1 strain and the *ku70^{nar1}* single mutant. Serial 6-fold dilutions of cultures of the same density were spotted on the plates, incubated at 30°C for 2 ½ days, and then photographed. **(c)** The indicated strains were first grown in liquid MMD culture, and then washed and resuspend in YPD such that OD_{600} = 0.05. The cultures were grown for 20 hours at 30^{0}C in YPD, and then examined under the microscope.

Supplementary Fig. 10

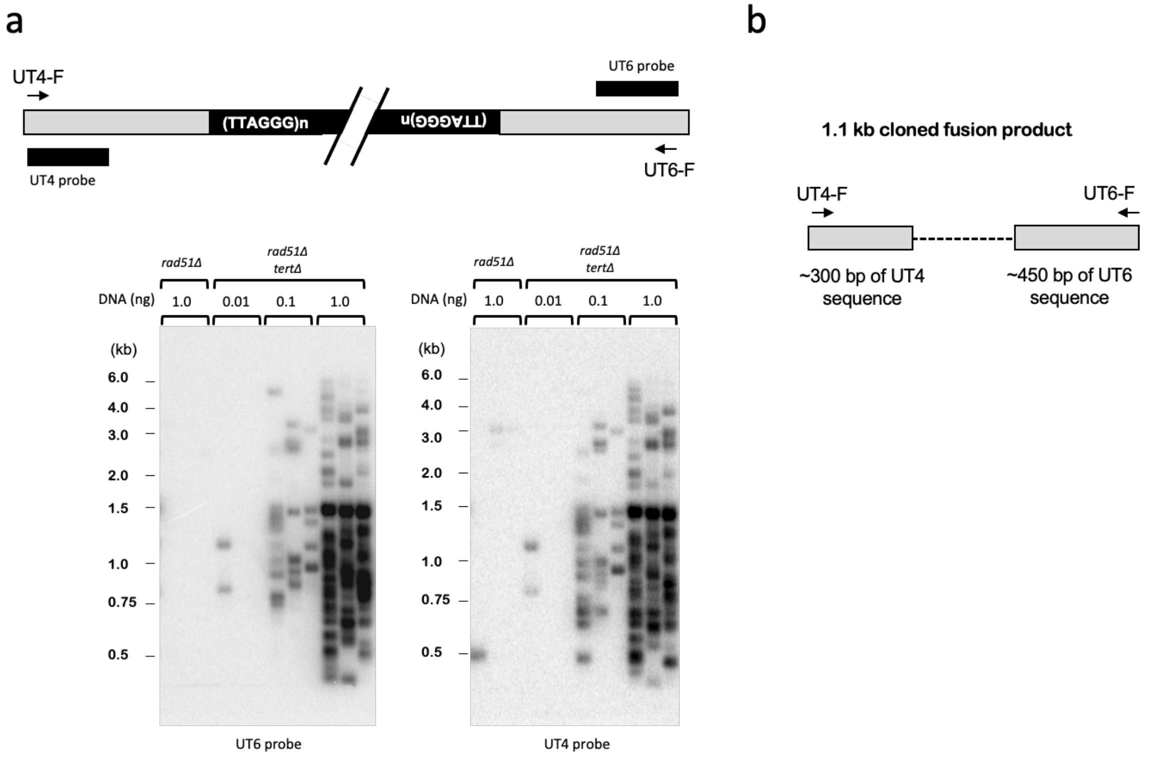

Supplementary Fig. 10. Detection of telomere-telomere fusions in Ustilago maydis .
(a) The fusions between UT-4 and UT-6 type telomeres in the indicated strains were amplified using primers that extend toward chromosome ends. The PCR products were detected using successively subtelomere probes from UT-6 and UT-4. The similarities in banding patterns between the two blots indicate that most of the PCR products contain both UT-4 and UT-6 sequences. **(b)** The pool of amplified fusion products from *rad51*Δ *tert*Δ were cloned into pMiniT 2.0 vector (NEB PCR cloning kit, New England Biolab Inc.), and a recombinant clone carrying a 1.1 Kb insert was sequenced from both ends. Consistent with telomere-telomere fusions, approximately 300 and 450 bp of readable sequences from the two ends matched sequences from the UT-4 and UT-6 subtelomeres, respectively. We were unable to sequence across the junction, which may contain long inverted repeats.

Supplementary Fig. 11

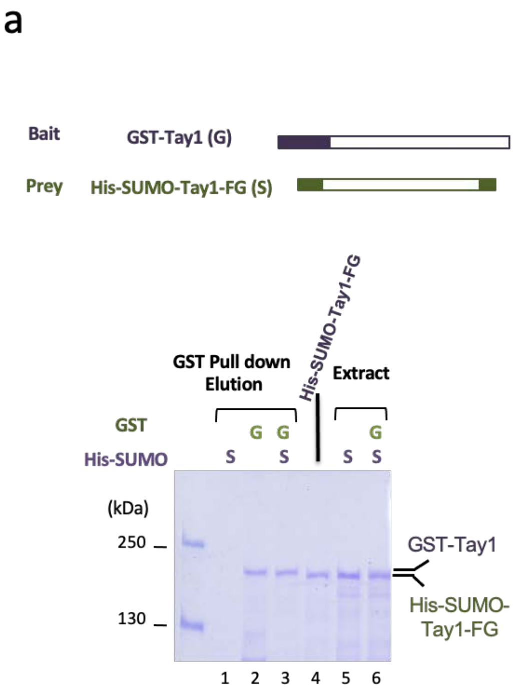

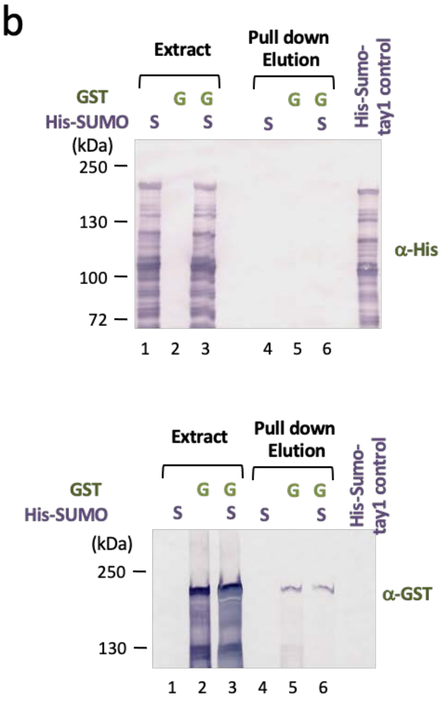

Supplementary Fig. 11. Analysis of potential interaction between GST-Tay1 and His-SUMO-Tay1-FG
(a) Differently tagged Tay1 proteins (GST-Tay1 and His-SUMO-Tay1-FG) were expressed separately or together in *E. coli* and subjected to glutathione-Sepharose pull down. The tagged proteins are illustrated schematically on top. The extracts and pull down samples were analyzed by SDS-PAGE and Coomassie staining, and the stained gel shown on the bottom. Note that the faster migrating His-SUMO-Tay1-FG was expressed at a much higher level than the slower migrating GST-Tay1, but could not be detected in the pull down samples (compare lanes 3 and 6), suggesting that the two proteins did not form a stable complex. **(b)** The indicated extracts and pull down samples were subjected to Western analysis using anti-HIS and anti-GST to detect His-SUMO-Tay1-FG and GST-Tay1, respectively. Note that even though there was abundant His-SUMO-Tay1-FG (both full length and proteolytic fragments) in the extracts, none could be detected in the pull down samples (compare lanes 3 and 6), again indicating that the two tagged Tay1s do not form a stable complex.

Supplementary Fig. 12

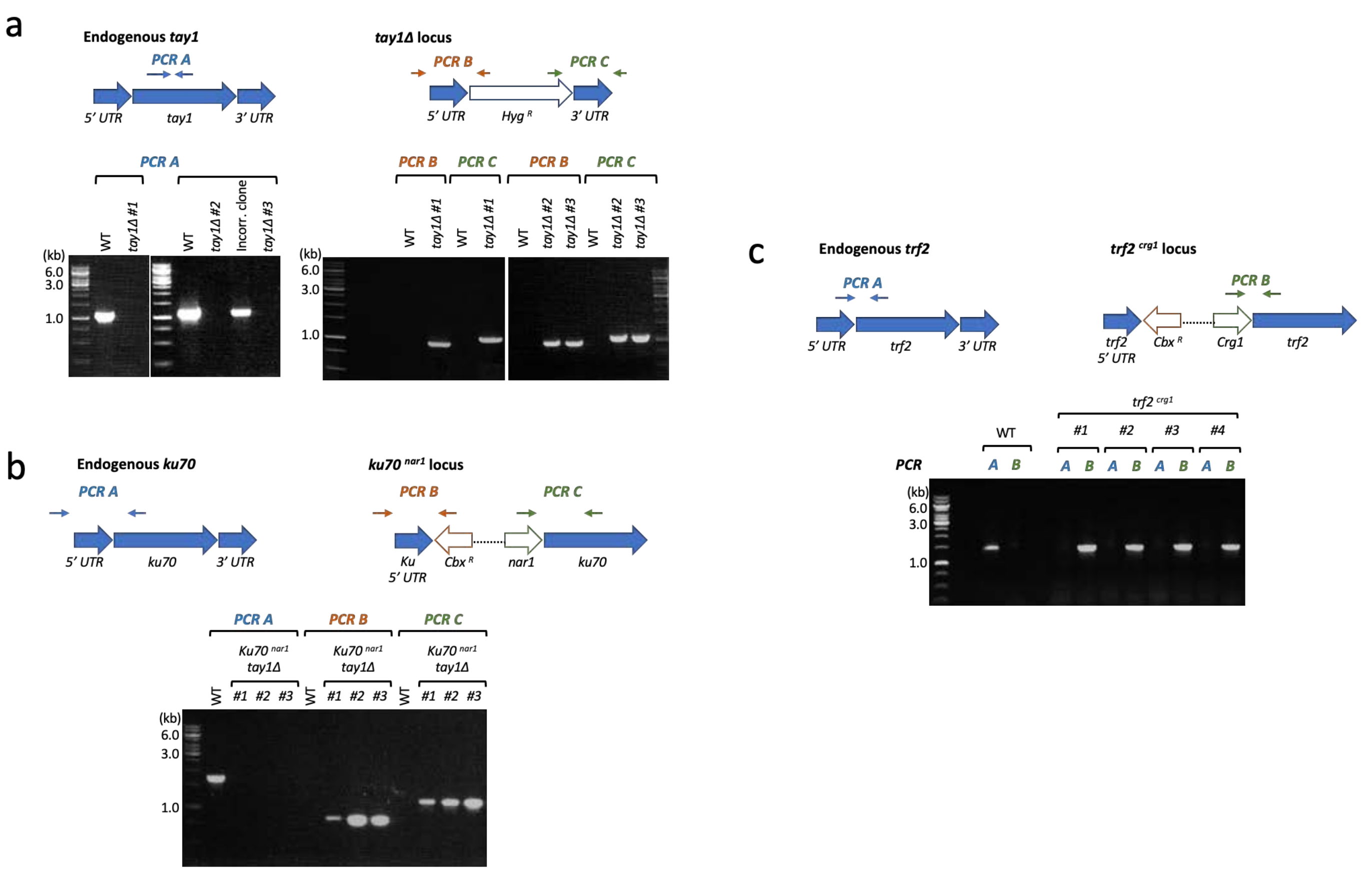

Supplementary Fig. 12. Genotyping of the *tay1*Δ, *$trf2^{crg1}$*, and *$ku70^{nar1}$ tay1*Δ mutants

(a) PCR-based genotyping of three independently derived *tay1*Δ mutants. The primers used were: UmTay1-F-Hind and UmTay1-1100-R for PCR A; UmTay1-EcoRI-F and Hygromycin-P1-R for PCR B; Hygromycin-P2-F and UmTay1-3UTR-BamHI-R for PCR C. **(b)** Genotyping of three independent *$ku70^{nar1}$ tay1*Δ mutants that were derived from a confirmed *tay1*Δ strain. The primers used include: UmKu70-5UTR-705-F and UmKu70-2100-R for PCR A; UmKu70-5UTR-705-F and Carboxin-P1 for PCR B; Pnar1-P1 and UmKu70-2100-R for PCR C. **(c)** Genotyping of four *$trf2^{crg1}$* mutants. The primers used include: UmTrf2-up-200 and UmTrf2-1100-R for PCR A; UmCrg_prom-200 and UmTrf2-1100-R for PCR B.

Supplementary Table S1. ***U. maydis*** **strains used in this study**

**Alias (Haploids)**	***Relevant Genotype***	**Reference**
FB1[a]	Wild type	(Banuett and Herskowitz, 1989)
UEY25 [ab]	*tay1Δ*	This work
UNFL100 [ac]	*blmΔ*	This work
UNFL101 [abc]	*tay1Δ blmΔ*	This work
UCS33	$uku70^{nar1}$	(de Sena-Tomas et al., 2015)
UEY26 [ab]	$uku70^{nar1}$ *tay1Δ*	This work
UCM350 [d]	wild type	Kojic et al., 2002
UEY15	*trt1Δ rad51Δ*	(Yu et al., 2018)
USZ100 [de]	$trf2^{crg1}$	This work
USZ101 [df]	$tay1^{crg1}$	This work
USZ102 [dg]	$trt1^{crg1}$	This work
USZ103 [dfg]	$tay1^{crg1}$ $trt1^{crg1}$	This work

[a] The genotype of FB1 is *a1b1* for the mating type loci.
[b] *tay1* was disrupted by the insertion of *hph* cassette expressing the hygromycin resistance gene (Hyg^R).
[c] *blm* was disrupted by the insertion of *cbx* cassette expressing the carboxin resistance gene (Cbx^R).
[d] The genotype of UCM350 *is nar1-6 pan1-1 a1b1*. *nar*, *pan*, and *ab* indicate inability to reduce nitrate, auxotrophic requirement for pantothenate, and mating type loci, respectively.
[e] *trf2* was placed downstream of the arabinose-dependent *crg1* promoter through the introduction of a Cbx^R-containing cassette.
[f] *tay1* was placed downstream of the arabinose-dependent *crg1* promoter through the introduction of a Cbx^R-containing cassette.
[g] *trt1* was placed downstream of the arabinose-dependent *crg1* promoter through the introduction of a Hyg^R-containing cassette.

Supplementary Table S2. Oligos used in this study

Name	Sequence 5' to 3'
**Protein Expression**	
UmTay1-F-Hind	AAT AAGCTT CT ATG CCG TCG CAT CCG CAA CCG GCT
UmTay1-R-FG-NotI	AAT GCGGCCGC CTA CTT GTC ATC GTC ATC CTT GTA ATC ATG CTG CTG CCG CTG CTG ATC
UmTay1-274-R-FG-NotI	AAT GCGGCCGC CTA CTT GTC ATC GTC ATC CTT GTA ATC GCG ATC CTT GCG TGC CTT GGA
UmBlm-F-Nco	AAT CCATGG CA ATG CCG CAA TCC GCA CTA ACC CCA
UmBlm-R-FG-NotI	AAT GCGGCCGC CTA CTT GTC ATC GTC ATC CTT GTA ATC ACC CGA ACG AGG TAG ATT GGG C
UmTrf2-F-SalI	ATTAC GTCGAC GT ATG TCA GCT TCT GCT CGG AGC
UmTrf2-416F-SalI	ATTAC GTCGAC CG CAA TCC GAA CAG CGG TTA
UmTrf2-dn-FG-NotI	AA GCGGCCGC TTA CTT GTC ATC GTC ATC CTT GTA ATC TTC GTT TGA AAG AGA ACT CGA CG
UmTrf2-1350-up-Sal	ATTAC GTCGAC GT ATG TCG GGA CGA GTA CGA TGG
UmTrf2-1349-dn-FG	AA GCGGCCGC TTA CTT GTC ATC GTC ATC CTT GTA ATC GTT GTG ACC AGA GAC GTA CAG
**Strain Construction and Genotyping**	
UmTrf2(1)Nde	GAAATC CAT ATG TCAGCTTCTGCTCGGAG
UmTrf2(700)Xba	ATT TCTAGA CATCCAGAGCCTCCTGGGA
UmTrf2(-700)Xba	ATA TCTAGA GCAGTCATCATTATTGCCAT
UmTrf2(-1)Eco	ATT GAATTC TGTGGCCCACACGTTACT
UmTrf2-up-200	CCAGAAGCAAAAAGAGCAGACG
UmTrf2-1100-R	TCTTCCGAGCCTTCATCCGA
UmTay1(1)Nde	GAAATC CAT ATG CCGTCGCATCCGCAA
UmTay1(699R)Xba	ATT TCTAGA CTTTGCGATGAGTGCCC
UmTay1(-700)Xba	ATA TCTAGA TGAGATTGCTTATTAGTTCG
UmTay1(-1R)Eco	ATT GAATTC ACTGGAGGCGATCGTGAG
UmTrt1(1)Nde	GAAATC CAT ATG CAGCCACCCAAATCCAG
UmTrt1(700R)AvrII	ATT CCTAGG CATGTTTGCGCTTAGCACCA
UmTrt1(-685)AvrII	ATA CCTAGG ATCTATCTCAACACCTCGCA
UmTrt1(-1R)Bam	ATT GGATCC CTCAGTTCTTCATGTTCTGA
UmCrg_prom-200	TGCAACATGAAGTTAGGTGTAGGC
Tay1-5UTR-Vector-F	CCG GGC CCC CCC TCG AGA ATT CGT CAG TGA AAG AAA AGT GTG AAG CAG TAA GTA TTC
Tay1-5UTR-Hyg-R	TGT CAC GCC ATG GT ACT GGA GGC GAT CGT GAG AGG CAA GTG GCA GAC GAG
Tay1-3UTR-Hyg-F	GCG GCC GCA TTA ATA CAG GCG CCT TCC GTC GAG TCT TAC TAC CAA TCA CG
Tay1-3UTR-Vector-R	GGG CGA ATT GGA GCT CGG ATC CAC CAA TCA AGC CGC TTG TGC CAC GCA CAA CAT GCC
Hyg-Tay1-5UTR-F2	GAT CGC CTC CAG TAC CAT GGC GTG ACA ATT GCG GCC GCA CTC GAG TG
Hyg-Tay1-3UTR-R2	GAA GGC GCC TGT ATT AAT GCG GCC GCA CAG CTT CGC GGC GCA GCA G
Vector-Tay1-3UTR-F	CAA GCG GCT TGA TTG GTG GATC CGA GCT CCA ATT CGC CCT ATA GTG AGT CGT ATT AC
Vector-Tay1-5UTR-R	CTT TTC TTT CAC TGA CGA ATT CTC GAG GGG GGG CCC GGT ACC AGC TTT TGT TCC C
UmTay1-F-Hind	AAT AAGCTT CT ATG CCG TCG CAT CCG CAA CCG GCT

UmTay1-1100-R	CCCATCGAGTTGGAGCGG
UmTay1-EcoRI-F	CCGGAATTCCAGTCTGTGAGTGGTGCG
Hygromycin-P1-R	GGTGCCCGAGAGAACAATTTTCCGATTGT
Hygromycin-P2-F	GCACTATATCATAGTGAAACTCGCAACAAC
UmTay1-3UTR-BamHI-R	CCGGGATCCCCACATCCACCGTTCGACTC
UmKu70-5UTR-705-F	GCCCAAATCCCAGAATGCGGG
UmKu70-2100-R	AGCCTTTCCTCGCCTTGGTC
Carboxin-P1	GCTGGTTCCAGCAGCCGATG
Pnar1-P1	GGCGTCATTTGATTCTCTCACGC
**Helicase Assays**	
NT-substrate-top	TTCTTCCTTTCCCTCTTCCTGATACGGCTGCTTCTCATCTACAACGTGATCCGTCATGGT
NT-substrate-bottom	ATGAGAAGCAGCCGTATCAGGAAGAGGGAAAGGAAGAA
Telo-substrate-top	TTCTTCCTTTCCCTC**TAGGGTTAGGGTTAGGGTTAGGGTTAGGGTTAGGGTTAGGGTTAG**
Telo-substrate-bottom	**CCCTAACCCTAACCCTAACCCTA**GAGGGAAAGGAAGAA
**PCR, hybridization, and EMSA assays**	
TTAGGG$_4$	TTAGGG TTAGGG TTAGGG TTAGGG
CCCTAA$_4$	CCCTAA CCCTAA CCCTAA CCCTAA
CCCTAA$_8$	CCCTAA CCCTAA CCCTAA CCCTAA CCCTAA CCCTAA CCCTAA CCCTAA
TTAGGG$_{3.5}$	TTAGGG TTAGGG TTAGGG TTAG
CCCTAA$_{3.5}$	CTAA CCCTAA CCCTAA CCCTAA
TTAGGG$_{2.5}$	TTAGGG TTAGGG TTAG
CCCTAA$_{2.5}$	CTAA CCCTAA CCCTAA
TTAGGG$_2$	GG TTAGGG TTAG
CCCTAA$_2$	CTAA CCCTAA CC
Yl TEL G-Strand	TTAGTCAGGG TTAGTCAGGG TTAGT
Yl TEL C-Strand	ACTAA CCCTGACTAA CCCTGACTAA
Cg TEL G-Strand	TGGGGTCTGGGTGCTG
Cg TEL C-strand	CAGCACCCAGACCCCA
UT4-2116-F	TCGGGCAACGTTCCATGTCG
UT6-2210-F	CTACTACACATCGGTTCAGGC

Supplementary Fig. 13. Original Gel and Blot images

Original images for Fig. 1b

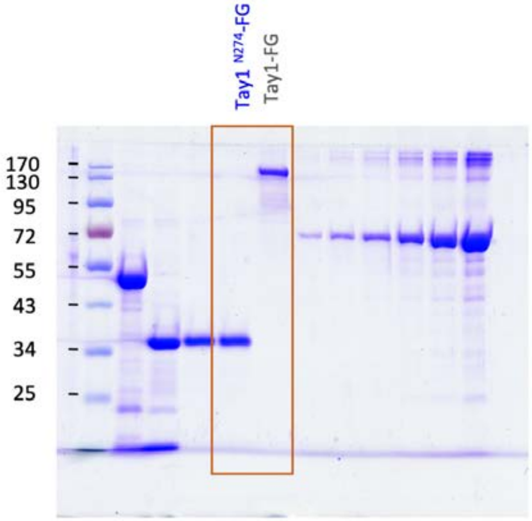

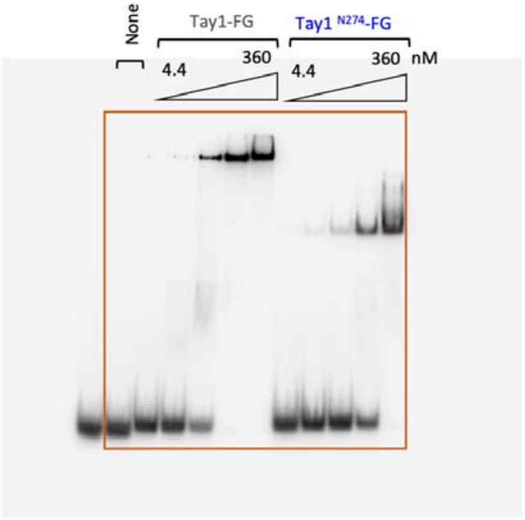

Original image for Fig. 1c

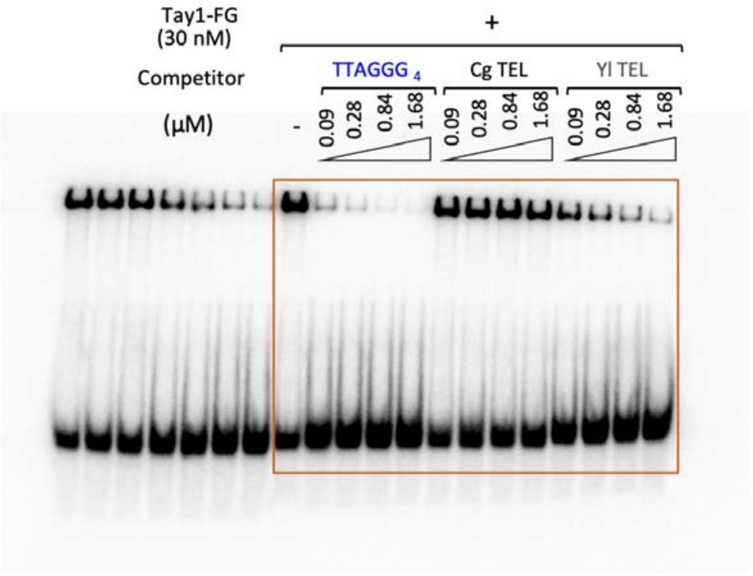

Original images for Fig. 2b and 2c

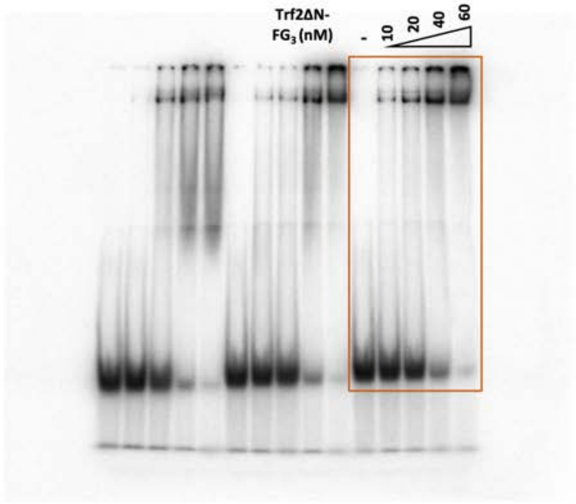

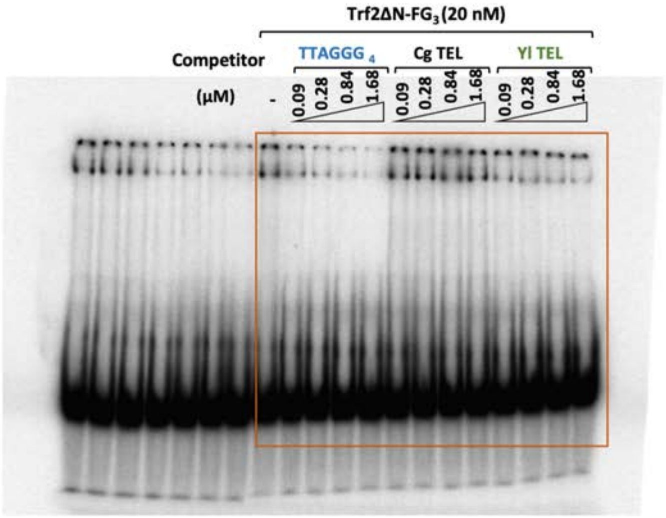

Original images for Fig. 3a and 3b

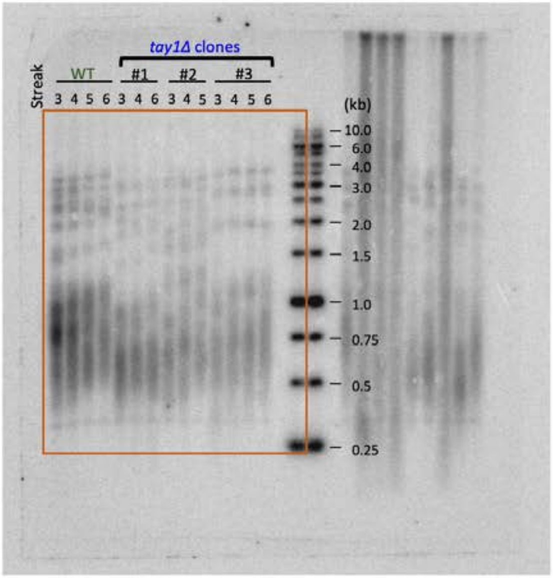

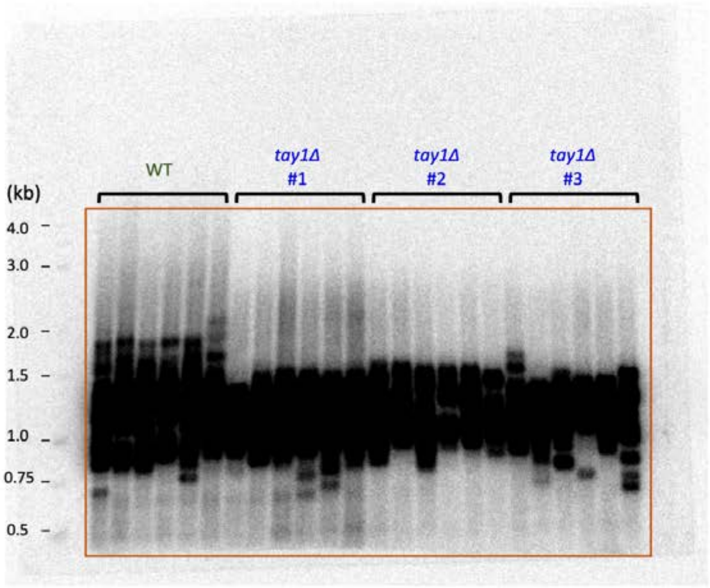

Original images for Fig. 4a and 4b

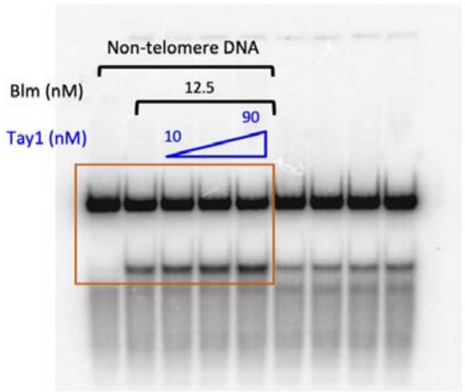

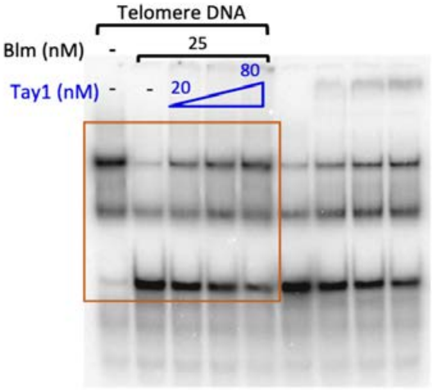

Original images for Fig. 4c and 4d

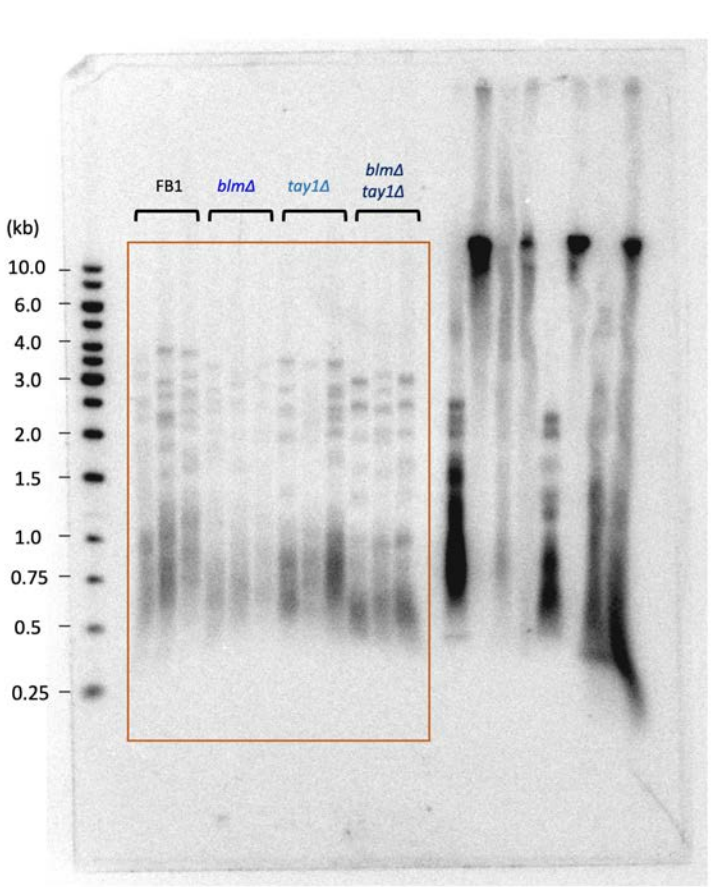

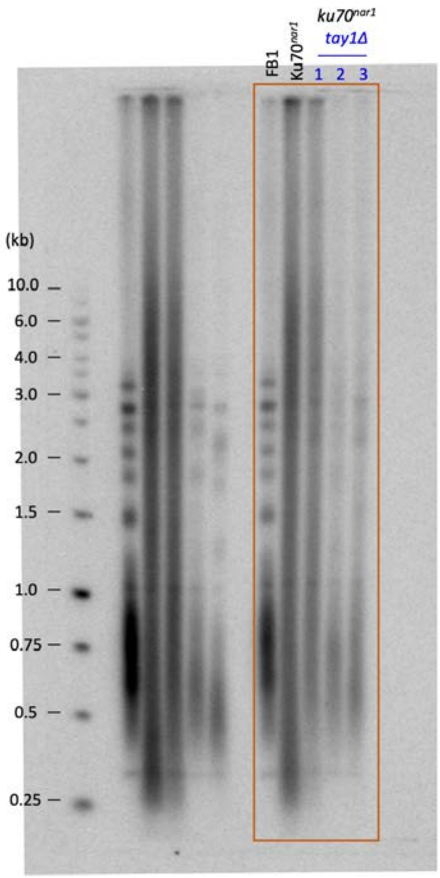

Original image for Fig. 4e

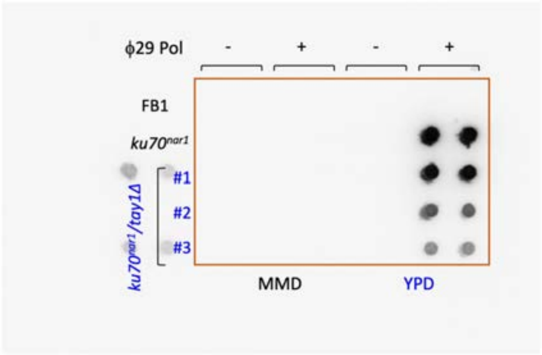

Original images for Fig. 5c and 5d

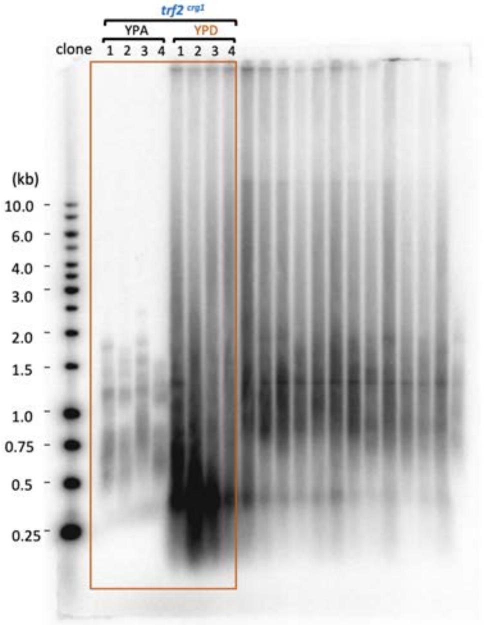

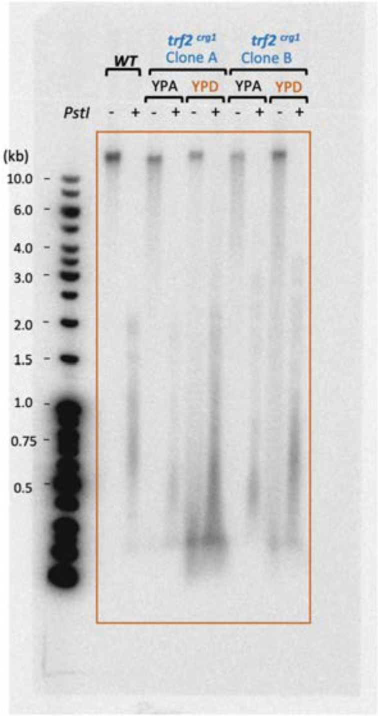

Original image for Fig. 5e

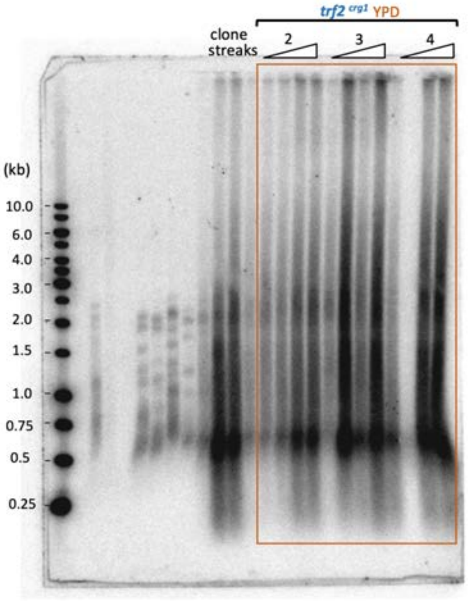

Original images for Fig. 6a

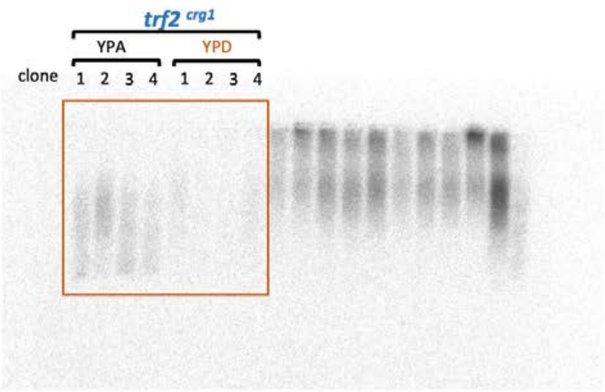

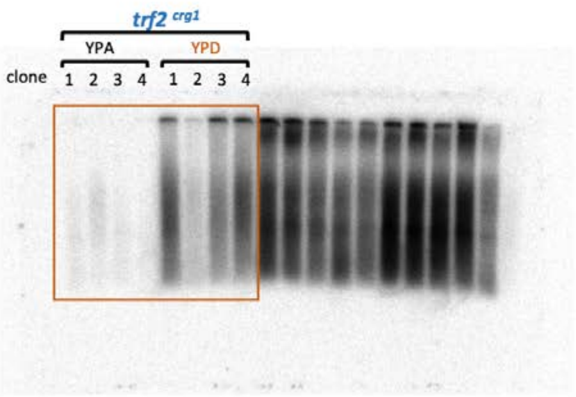

Original image for Fig. 6b

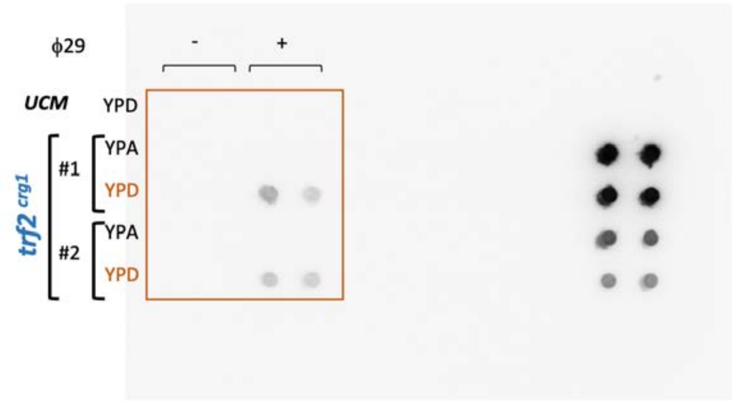

Original image for Fig. 6c

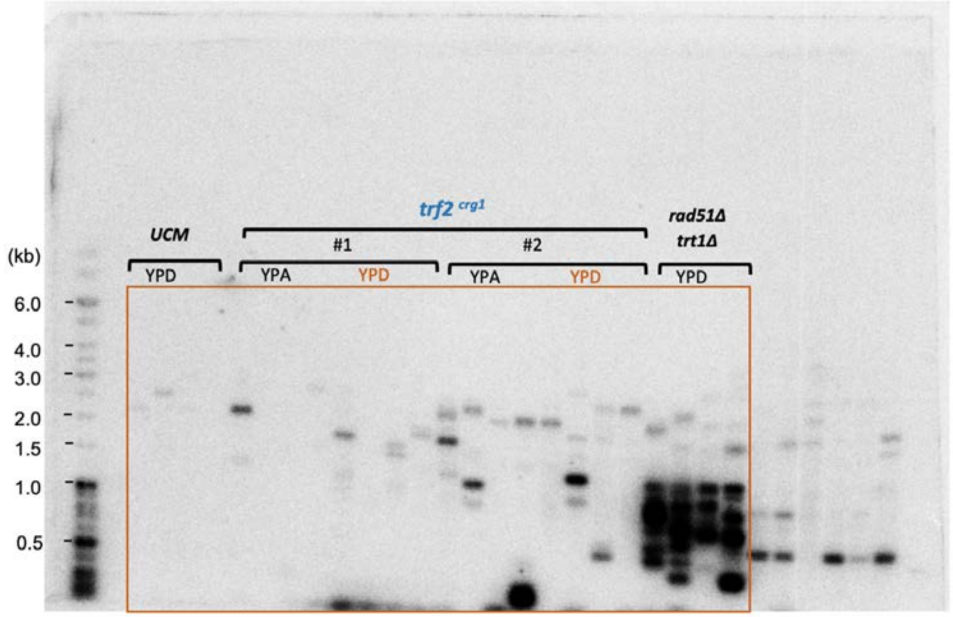

Supplementary References

1. Banuett, F., and Herskowitz, I. (1989). Different a alleles of Ustilago maydis are necessary for maintenance of filamentous growth but not for meiosis. Proceedings of the National Academy of Sciences of the United States of America *86*, 5878-5882.

2. Court, R., Chapman, L., Fairall, L., and Rhodes, D. (2005). How the human telomeric proteins TRF1 and TRF2 recognize telomeric DNA: a view from high-resolution crystal structures. EMBO Rep *6*, 39-45.

3. de Sena-Tomas, C., Yu, E.Y., Calzada, A., Holloman, W.K., Lue, N.F., and Perez-Martin, J. (2015). Fungal Ku prevents permanent cell cycle arrest by suppressing DNA damage signaling at telomeres. Nucleic acids research *43*, 2138-2151.

4. Lai, T.P., Zhang, N., Noh, J., Mender, I., Tedone, E., Huang, E., Wright, W.E., Danuser, G., and Shay, J.W. (2017). A method for measuring the distribution of the shortest telomeres in cells and tissues. Nat Commun *8*, 1356.

5. Yu, E.Y., Hsu, M., Holloman, W.K., and Lue, N.F. (2018). Contributions of recombination and repair proteins to telomere maintenance in telomerase-positive and negative Ustilago maydis. Mol Microbiol *107*, 81-93.
