# Supplementary material for: Structurally distinct telomere-binding proteins in Ustilago maydis execute non-overlapping functions in telomere replication, recombination, and protection
Source: Commun Biol. 2020 Dec 16;3:777. doi: 10.1038/s42003-020-01505-z (PMC7744550; doi:10.1038/s42003-020-01505-z)
Supplement: Supplementary file 3 — Description of Additional Supplementary Files [file 42003_2020_1505_MOESM3_ESM.docx]

Description of Additional Supplementary Files

**File Name:** Supplementary Data 1

**Description:** The original numerical data for all the plots in the Figures and Supplementary Figures.
